# Supplementary material for: Challenges and opportunities of the full phase-out of fossil fuels under the 1.5 °C goal
Source: Nat Commun. 2026 May 18;17:4379. doi: 10.1038/s41467-026-72841-7 (PMC13183887; doi:10.1038/s41467-026-72841-7)
Supplement: Supplementary file 1 — Supplementary Information [file 41467_2026_72841_MOESM1_ESM.pdf]

Supplementary Information

Challenges and opportunities of the full phase-out of fossil fuels under the 1.5°C goal

Author list

Shotaro Mori[1,2]*, Siddharth Joshi[2], Volker Krey[2], Ken Oshiro[3], Oliver Fricko[2], Takuya Hara[2,4] and Shinichiro Fujimori[1,2,5]*

Affiliations

1. Kyoto University, C1-3, Kyotodaigaku-Katsura, Nishikyo-ku, Kyoto, Japan
2. International Institute for Applied System Analysis (IIASA), Laxenburg, Austria
3. Hokkaido University, Sapporo, Japan
4. Toyota Motor Corporation, Toyota, Aichi, Japan
5. National Institute for Environmental Studies (NIES), Tsukuba, Japan

* Corresponding author
Shotaro Mori: mori.shotaro.2n@kyoto-u.ac.jp
Shinichiro Fujimori: fujimori.shinichiro.8a@kyoto-u.ac.jp

Supplementary Figures

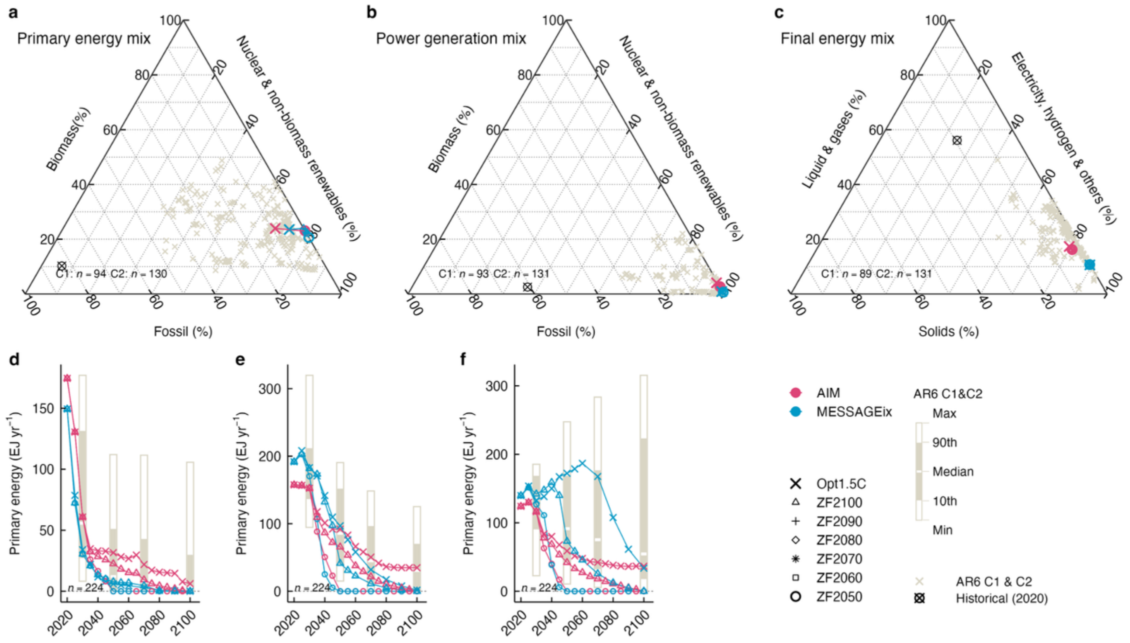

Supplementary Fig. 1 | Fossil fuel phase-out and energy system transformation. **a–c**, Energy mix for primary energy (**a**), power generation (**b**), and final energy (**c**) in 2100 shown as ternary diagrams. In these diagrams, the share of each component should be read from ticks parallel to the edge where that component equals zero. Coloured symbols represent energy mixes by scenario and model. Grey symbols represent the energy mixes of the Intergovernmental Panel on Climate Change Sixth Assessment Report (IPCC AR6) C1 and C2 scenarios for 2100. Black symbols represent the historical energy mix for 2020, based on the International Energy Agency (IEA) energy balance[1]. **d–f**, Annual primary energy supplies from coal (**d**), crude oil (**e**) and natural gas (**f**) from 2020 to 2100. Box plots illustrate primary energy supply from fossil fuels in IPCC AR6 C1 and C2 scenarios in 2030, 2050, 2070 and 2100. "*n*" denotes the number of available scenarios in each category. In this study, primary energy accounting was based on the direct equivalent method, which systematically reduces the contribution of non-combustible energy sources such as hydro, nuclear, solar, and wind energy compared to combustible fuels[2].

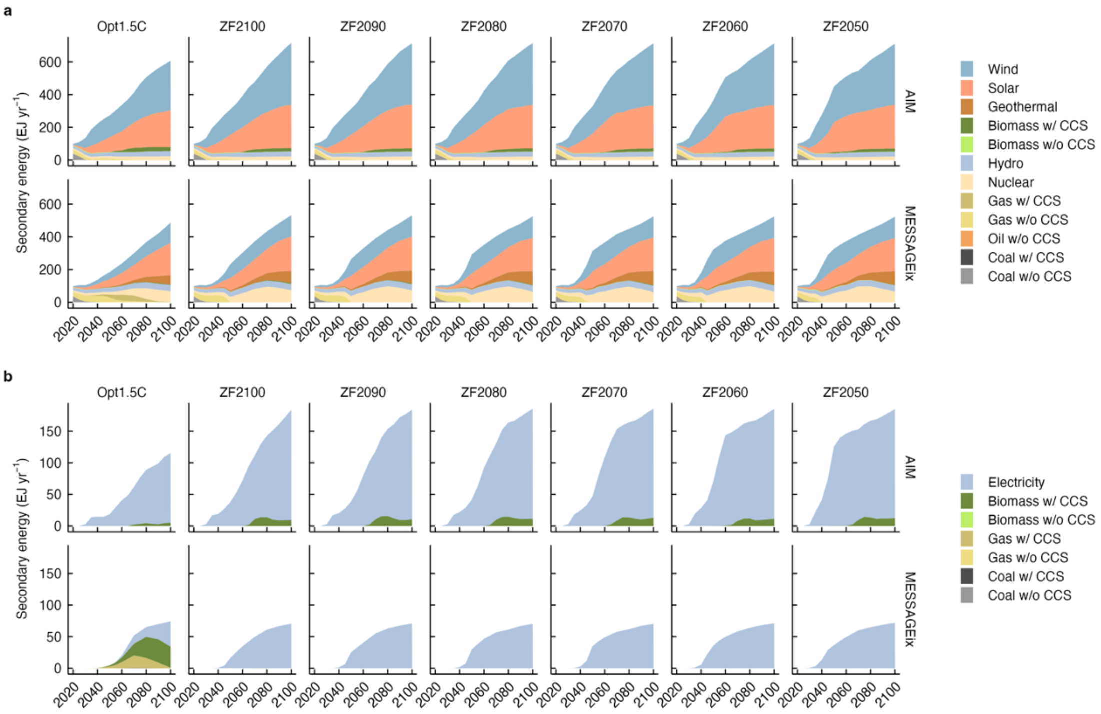

Supplementary Fig. 2 | Power and hydrogen generation. **a**, Power generation during the period 2020–2100. **b,** Hydrogen generation during the period 2020–2100.

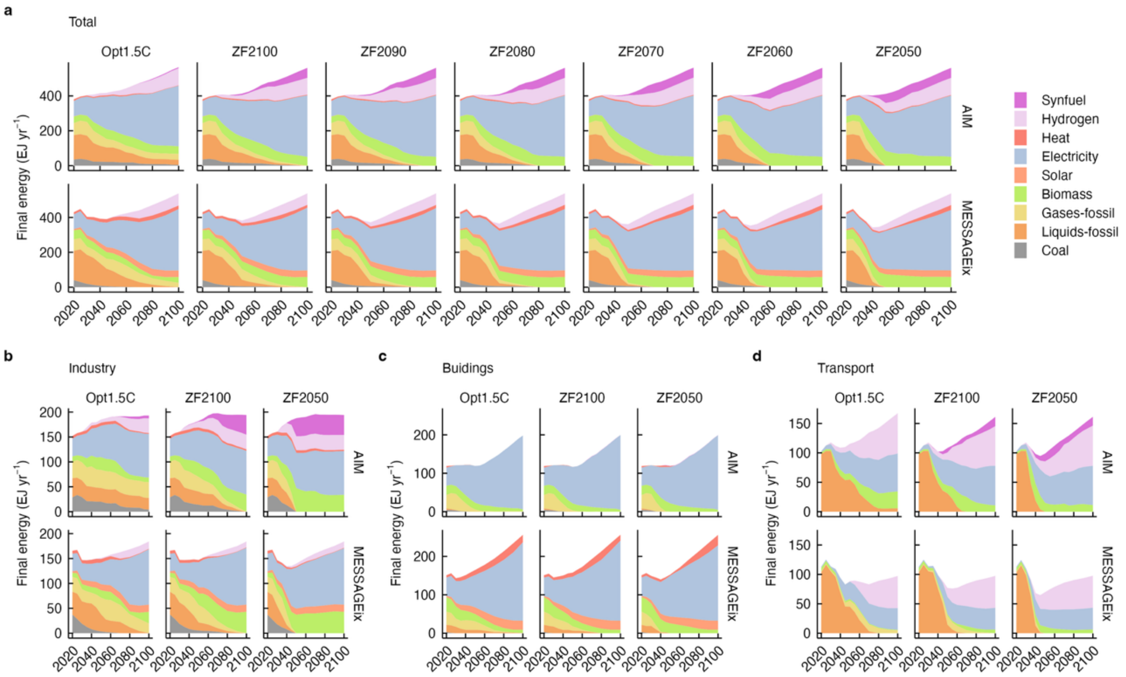

Supplementary Fig. 3 | Transformation to ZF energy systems in energy demand sectors. **a**, Final energy consumption during the period 2020–2100. **b–d**, Final energy consumption in industry (**b**), buildings (**c**) and transportation (**d**) sectors in the Opt1.5C, ZF2100 and ZF2050 scenarios during the period 2020–2100.

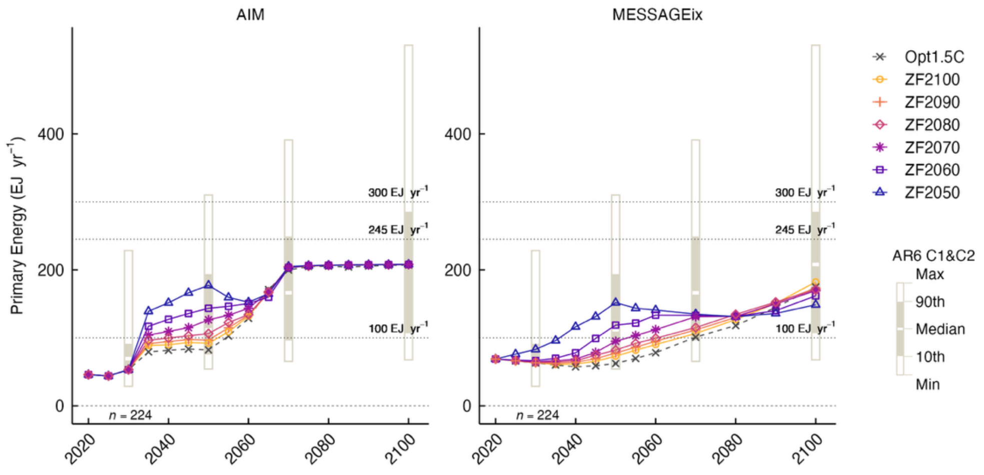

Supplementary Fig. 4 | Primary energy supply from biomass. Primary energy supply from biomass during the period 2020–2100. Creutzig et al. (2015)[4] derived an upper limit of 100–300 EJ/yr for sustainable biomass use, while IPCC AR6 WGIII[5] identified thresholds of 100 EJ/yr for medium concern and 245 EJ/yr for high concern from a sustainability perspective. Box plots illustrate primary energy supply from biomass in the IPCC AR6 C1 and C2 scenarios for 2030, 2050, 2070, and 2100. "*n*" denotes the number of available scenarios in each category.

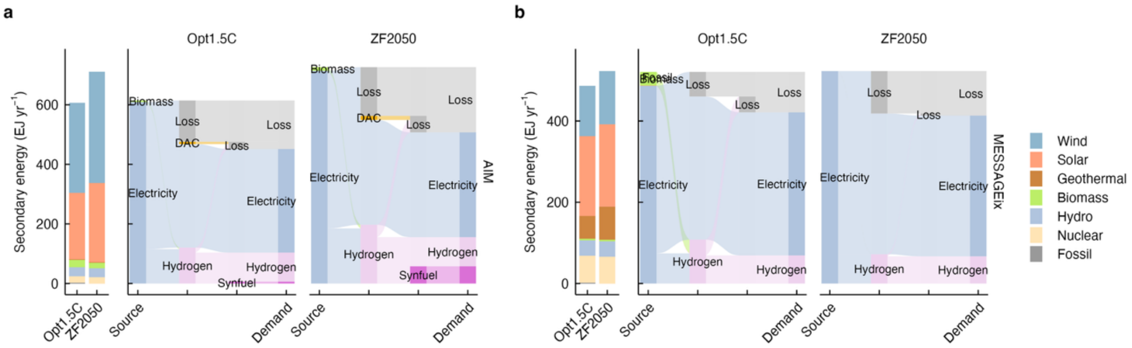

Supplementary Fig. 5 | Energy system transformation in energy supply sectors driven by a full phase-out of fossil fuels. a, b, Power generation mixes and secondary energy flow in the Opt1.5C and ZF2050 scenarios of AIM-Technology (**a**) and MESSAGEix-GLOBIOM (**b**) in 2100.

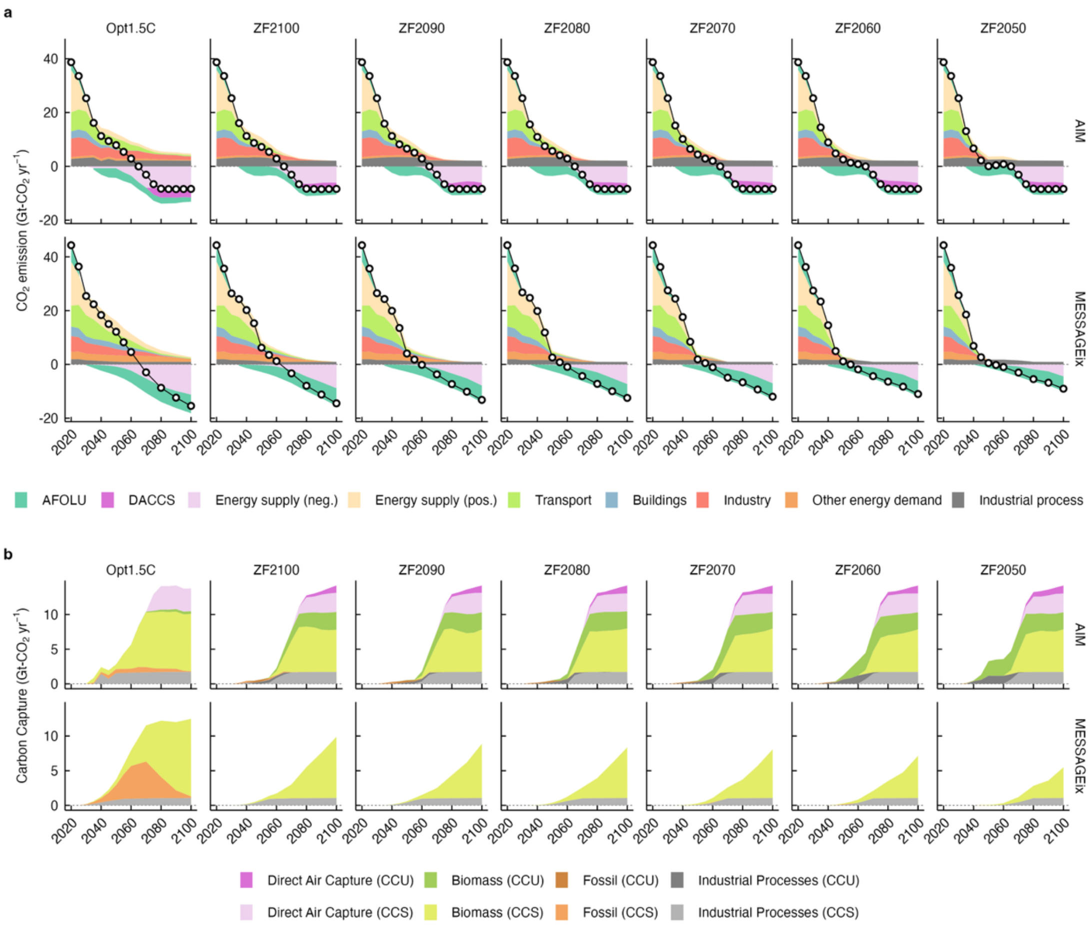

Supplementary Fig. 6 | CO_2 emissions and carbon capture, utilization and storage. **a**, Sectoral CO_2 emissions during the period 2020–2100. Line and dot plots show net CO_2 emissions from energy and AFOLU sectors during the period 2020–2100. **b**, Carbon capture and its destination, including CCU and CCS during the period 2020–2100.

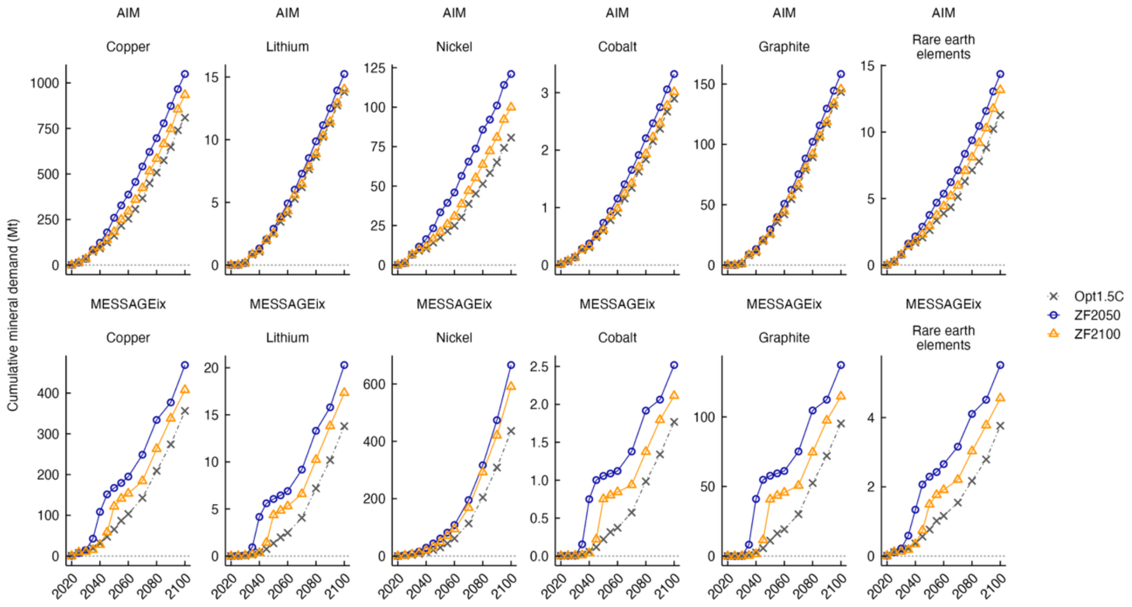

Supplementary Fig. 7 | Key mineral demand from energy supply technologies. Cumulative demand of key minerals, including copper, lithium, nickel, cobalt, graphite and rare earth elements (praseodymium, neodymium, terbium, and dysprosium), in the ZF2050, ZF2100 and Opt1.5C scenarios. The estimates cover mineral demands from 13 power generation technologies (coal with/without CCS, oil with/without CCS, gas with/without CCS, nuclear, hydro, geothermal, biomass with/without CCS, solar, and wind), as well as electricity storage and electrolysers. Mineral demand was estimated using the technology-specific mineral intensities (e.g., t GW^{-1}) reported in Wei et al. (2025)[3] together with the capacity additions (e.g., GW yr^{-1}) in each scenario. Further details of the methodology are provided in Supplementary Note 1.

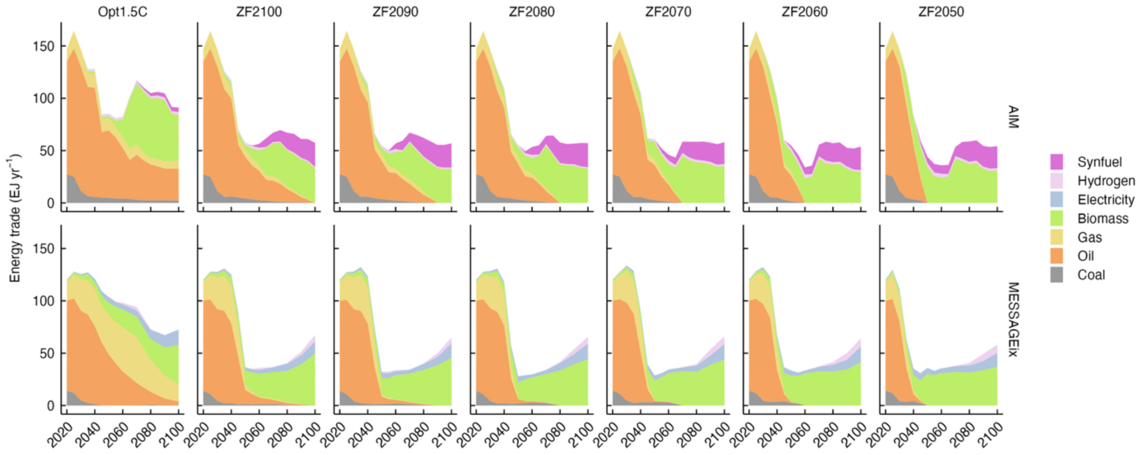

Supplementary Fig. 8 | Energy trade. International energy trade during the period 2020–2100. Hydrogen includes trade via liquefied hydrogen, methylcyclohexane (MCH), and ammonia.

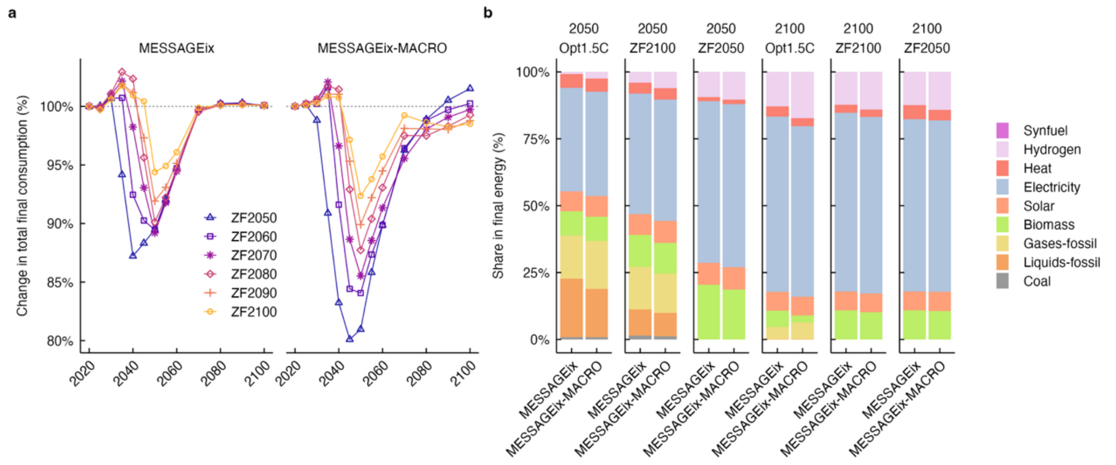

Supplementary Fig. 9 | Macroeconomic response of final energy demand in MESSAGEix-MACRO. a, Percentage change in total final consumption in the ZF scenarios relative to the Opt1.5C scenario, for the MESSAGEix standalone and MESSAGEix-MACRO model. **b,** Final energy mix in 2050 and 2100 under the Opt1.5C, ZF2050, and ZF2100 scenarios in the MESSAGEix standalone and MESSAGEix-MACRO model. For details of the MESSAGEix-MACRO model, see Methods.

Supplementary Notes

Supplementary Note 1 | Estimation of key mineral demand

We estimated the demand for key minerals, including copper, lithium, nickel, cobalt, graphite, and rare earth elements (praseodymium, neodymium, terbium and dysprosium), following the data and approach of Wei et al. (2025)[3], as shown in Supplementary Fig. 7. The coverage of these key minerals was defined in accordance with the key energy transition minerals specified in the IEA's Global Critical Mineral Outlook 2025[6]. Wei et al. (2025)[3] provide technology-specific mineral intensity data (e.g., t GW^{-1}) for 13 power generation technologies (coal with/without CCS, oil with/without CCS, gas with/without CCS, nuclear, hydro, geothermal, biomass with/without CCS, solar, and wind) as well as for electricity storage and electrolysers. For solar power, electricity storage, and electrolysers, the data are further disaggregated into multiple technology types, and individual mineral intensities and future market shares are provided for each. By combining these mineral intensities with annual capacity additions (e.g., GW yr^{-1}), we estimated the corresponding mineral demand (e.g., t yr^{-1}) for each scenario. It should be noted that, as discussed as one of the limitations in Wei et al. (2025)[3], their technology-specific mineral intensity data do not cover several technologies such as electric vehicles and transmission grids. In addition, this study does not account for mineral recycling, and therefore the estimated mineral demand represents the total amount, including the portion that could be supplied by recycled minerals.

Supplementary References

1. IEA. *World Energy Balances*. (OECD/IEA, Paris, 2022).
2. IPCC. Annex II: Definitions, Units and Conventions [Al Khourdajie, A., R. van Diemen, W.F. Lamb, M. Pathak, A. Reisinger, S. de la Rue du Can, J. Skea, R. Slade, S. Some, L. Steg (eds)]. in *IPCC, 2022: Climate Change 2022: Mitigation of Climate Change. Contribution of Working Group III to the Sixth Assessment Report of the Intergovernmental Panel on Climate Change* (eds Shukla, P. R. et al.) (Cambridge University Press, Cambridge, UK and New York, NY, USA, 2022). doi:10.1017/9781009157926.021.
3. Wei, Y.-M. et al. Navigating energy transition solutions for climate targets with minerals constraint. *Nat. Clim. Change* **15**, 833–841 (2025).
4. Creutzig, F. et al. Bioenergy and climate change mitigation: an assessment. *GCB Bioenergy* **7**, 916–944 (2015).
5. IPCC. Annex III: Scenarios and modelling methods [Guivarch, C., E. Kriegler, J. Portugal-Pereira, V. Bosetti, J. Edmonds, M. Fischedick, P. Havlík, P. Jaramillo, V. Krey, F. Lecocq, A. Lucena, M. Meinshausen, S. Mirasgedis, B. O'Neill, G.P. Peters, J. Rogelj, S. in *IPCC, 2022: Climate Change 2022: Mitigation of Climate Change. Contribution of Working Group III to the Sixth Assessment Report of the Intergovernmental Panel on Climate Change* (eds Shukla, P. R. et al.) (Cambridge University Press, Cambridge, UK and New York, NY, USA, 2022). doi:10.1017/9781009157926.022.
6. IEA. *Global Critical Minerals Outlook 2025*. (2025).
